# Measuring Strengths, Opportunities, Aspirations, and Results: Psychometric Properties of the 12-Item SOAR Scale

**DOI:** 10.3389/fpsyg.2022.854406

**Published:** 2022-04-08

**Authors:** Matthew L. Cole, Jacqueline M. Stavros, John Cox, Alexandra Stavros

**Affiliations:** ^1^College of Business and Information Technology, Lawrence Technological University, Southfield, MI, United States; ^2^Walsh College, Troy, MI, United States

**Keywords:** SOAR scale, psychometric properties, bifactor analysis, measurement invariance, exploratory structure equation modeling

## Abstract

Strengths, Opportunities, Aspirations, and Results (SOAR) is a strengths-based framework for strategic thinking, planning, conversations, and leading that focuses on strengths, opportunities, aspirations, and results. The SOAR framework leverages and integrates Appreciative Inquiry (AI) to create a transformation process through generative questions and positive framing. While SOAR has been used by practitioners since 2000 as a framework for generating positive organizational change, its use in empirical research has been limited by the absence of reliable and valid measures. We report on the reliability, construct validity, and measurement invariance of the SOAR Scale, a 12-item self-report survey organized into four first-order factors (Strengths, Opportunities, Aspirations, and Results). Data from a sample of 285 U.S. professionals were analyzed in Mplus using confirmatory factor analysis and exploratory structural equation modeling. The Four-Factor first-order exploratory structure equation modeling (ESEM) had the best model fit. Measurement invariance tests found the scalar invariance of the SOAR Scale across gender and education groups. Implications are discussed for using the SOAR Scale to build resilience at the individual, the team, and the organizational levels.

## Introduction

Since 2003, there has been a body of research that focuses on the positive states of individuals in organizational life that has influenced the social sciences called positive organizational scholarship (POS) (Cameron et al., 2003; Cameron and Spreitzer, 2012). This research places attention on enablers (e.g., strengths), motivators (e.g., aspirations and opportunities), and outcomes (e.g., results) (Cameron et al., 2003). Sekerka et al. (2014) found that the POS research is focused on describing, explaining, and predicting what types of thinking and behaving are associated with the best of what can be. The POS discipline of research has been drawn from various bodies of literature in several fields such as positive psychology, community psychology, Appreciative Inquiry (AI), and organization development. The focus of positive psychology is on positive traits and experiences (Seligman and Csikszentmihalyi, 2000). This body of research seeks to understand what represents the best of human conditions (strengths) and possibilities (opportunities). A recent study by McKinsey and Company suggests that capability building programs that focus on what employees are doing well (strengths) and support ways to learn more and to do even better (opportunities) can power continuous improvement, productivity, and organizational transformation. This is done *via* a “well designed program to promote productive behavior and skills [that] can not only energize an organization’s workforce but also become an essential element of any successful organization transformation” (Bachmann et al., 2021, p. 1).

Research confirms that positive emotions resulting from a focus on strengths and opportunities promote the success of an individual and team performance in organizations (Fredrickson, 2003a, 2009a; Miglianico et al., 2020; Dubreuil et al., 2021). A McKinsey Quarterly study of 1,300 global executives echoes this sentiment; in that, the highest performing organizations have a clear purpose, an understanding of strengths, shared aspirations, and leaders who know how to unleash ideas (opportunities) with a result-driven process (Isern and Pung, 2007). Empirical research by POS confirms strengths-based practices, and positive leadership improves the organizational performance and the individual physiological health and wellbeing (Tombaugh, 2005; Burkus, 2011; Cameron, 2013; Welch et al., 2014; Ding et al., 2020).

The other discipline is organization development (OD), which was founded on behavioral aspects of leading transformation and change, especially with a focus on AI. AI is a strengths-based philosophy with an organizational change approach that builds on the strengths of what is giving life to the organization---its positive core. It was co-created by its thought-leaders David Cooperrider and Ron Fry from the Case Western Reserve University in the late 1980s^1^. AI is focusing on the best in people, their organizations, and the relevant world around them (Cooperrider and Whitney, 2000). Additionally, it has become an effective theory and practice for transformation and change through the use of generative questions that can lead to positive images, positive actions, and positive outcomes in human systems in organizations (Whitney and Trosten-Bloom, 2003). A large body of empirical research has shown that AI creates upward spirals of positive emotions in people and organizations that increase appreciative behaviors and psychological wellbeing, capital, and safety (Fredrickson, 1998, 2003b,^2004^, 2009b; Verleysen et al., 2015; Daulon et al., 2017; Holma et al., 2017; Edmondson, 2019). AI research supports high performing employees who are more positive and have strengths based in nature, ask appreciative questions, and are more focused outside of themselves to help others (Brunetto et al., 2019; Peláez et al., 2020).

A related construct to AI is SOAR (Strengths, Opportunities, Aspirations, and Results), a strengths-based framework for strategic thinking, planning, conversations, and leading that integrates the theory and practice of POS, positive psychology, and AI. SOAR leverages the two AI practices of generative questions and positive framing (Cole and Stavros, 2019). While SOAR has been used by practitioners over the past two decades as a framework for building strategic capacity, psychological capacity, and resilience at the individual, the team, and the organizational level, its use in empirical research has been limited by the absence of a reliable and a valid measure of SOAR (Stavros and Saint, 2010; Stavros, 2020). We report on the psychometric properties of the SOAR Scale, a brief 12-item survey organized into four first-order factors (Strengths, Opportunities, Aspirations, Results).

## The Strengths, Opportunities, Aspirations, and Results Framework

The SOAR framework enhances strategic thinking, planning, conversations, and leading through a generative approach to inquire into strengths, opportunities, aspirations, and measurable results to shape a preferred future, allowing for positive changes in strategies, structures, business models, systems, people, and processes (Stavros et al., 2003; Stavros and Hinrichs, 2009; Stavros and Saint, 2010; Cole and Stavros, 2019; Stavros, 2020). SOAR has been contrasted to the classic SWOT diagnostic analysis that diverts organizational resources away from strengths and opportunities by a focus on weaknesses and threats. Rather, SOAR is a dialogue-based, whole system approach to OD that leverages generative dialogue and interactive communication to focus energy on aspirations and results (Stavros and Cole, 2013; Bushe and Marshak, 2014). The SOAR framework transforms strategic thinking, planning, conversations, and leading through the use of generative questions designed to leverage the capacity for positive change through stories and conversations about what works and organizational perspectives from relevant stakeholders (Stavros and Wooten, 2012; Bushe, 2013; Stavros and Torres, 2018). SOAR-based generative questions prompt reflection and divergent thinking, active listening, and collaboration to help stakeholders reframe reality in novel and creative ways (Stavros et al., 2003).

## The Strengths, Opportunities, Aspirations, and Results Scale

To help individuals learn and understand their natural capacity for strategic thinking, planning, conversations, and leading from a SOAR-based, generative perspective, we have developed the SOAR Scale, a 12-item self-report survey organized into four first-order factors representing the four elements of SOAR: Strengths, Opportunities, Aspirations, and Results. Capacity is the ability or potential to mobilize resources and create action to achieve objectives. It provides all that is necessary to construct relationships and develop capabilities needed to achieve both the individual and the organization’s values, vision, mission, goals, and strategy (Stavros, 1998). Strategic thinking is an intentional and a holistic way of thinking to create a strategy or strategic plan with a focus on desirable outcomes (Liedtka, 1998; Stavros and Hinrichs, 2009). Strategic thinking identifies organizational purpose and goals, builds relationships to drive the organization toward its purpose and goals, and identifies leverage points for organizational change. Strategic planning is “a complex cognitive, behavioral, social and political practice in which thinking, acting, learning, and knowing matter, and in which some associations are reinforced, others are created, and still others are dropped in the process of formulating and implementing strategies and plans” (Bryson et al., 2009, p. 176). A strategic plan includes the organization’s values, vision, mission, objectives, internal and external assessments of its environment, strategies, and implementation (action plan and resources to allocate). Strategic leading involves both thinking strategically and making strategic plans (Christensen, 1997).

### Items on the Strengths, Opportunities, Aspirations, and Results Scale

#### Strengths Factor (Three Items): Strengths, Assets, Capabilities

The first factor of the SOAR framework is Strengths. Strengths refer to a person’s natural capacity for optimal functioning and performance to attain value outcomes (Linley and Harrington, 2006). The strengths movement in positive psychology operates from the tenet that identification of strengths, and the use and application of strengths fosters optimal functioning and performance in various settings (Seligman et al., 2005; Harzer and Ruch, 2013; Dubreuil et al., 2021). Focusing on strengths means identifying and building on strengths as opposed to identifying and correcting weaknesses (Seligman, 2002; Clifton and Harter, 2003). Research shows that strengths-based interventions have positive outcomes for individuals and groups in the areas of wellbeing, work performance, work engagement, and organizational citizenship behaviors (Park et al., 2004; Littman-Ovadia and Steger, 2010; Lavy and Littman-Ovadia, 2016; Ghielen et al., 2018; Miglianico et al., 2020). The strengths factor in SOAR is defined as natural capacities, assets, or capabilities in self and others that allow optimal performance. As a strengths-based approach, SOAR builds on the work of these researchers through the identification of strengths as the starting point for a strategic conversation.

Strengths are also conceptualized in the literature as assets and capabilities that are naturally occurring and stable. Assets are those competencies that create positive outcomes and values at the individual, the team, and the organizational levels (Tedeschi and Kilmer, 2005; Park and Peterson, 2006; Brownlee et al., 2013; Frieden, 2019; Soares et al., 2019). Capabilities integrate, build, and reconfigure individual and organizational strengths to address changing environments that are superior to those of the competition (Trivette et al., 1990; Teece et al., 1997; Helfat and Peteraf, 2003; Wooten and Crane, 2004). Taken together, assets and capabilities offer the foundation for discovering and aligning an organization’s best strengths to a process of focusing on a stronger competitive advantage and a more sustainable future.

Several tools have been developed to assess strengths individually and in terms of assets and capabilities. Self-report tools to measure identification and the use of strengths include the Clifton Strengths-Finder (Rath, 2007), the Values in Action Inventory of Strengths (Park and Peterson, 2006), the Personal Strengths Inventory (Kienfie Liau et al., 2010), the Employee Strengths at Work Scale (Bhatnagar, 2020), and the Strengths Use Scale (Govindji and Linley, 2007). The Developmental Assets Profile (Scales, 2011) is a self-report inventory comprised of 58 strength items (Scales, 2011). Finally, the Family Functioning Style Scale (FFSS) is a 26-item self-report scale developed to measure individual strengths and capabilities along a five-point rating scale (Trivette et al., 1990; Danışman and Tiftik, 2014).

The SOAR Scale combines self-reported identification of and use of strengths, assets, and capabilities in one tool *via* three items that measure the first-order factor, Strengths, along a five-point rating scale (“never” to “always”). Each item is preceded by the phrase “When you think strategically, how often do you focus on…” Strengths (those natural capacities in self and others that allow optimal performance), Assets (those competencies that create personal, team, or organizational value), and Capabilities (those abilities that create the best for yourself, your team, or organization).

#### Opportunities Factor: Opportunities, Ideas, Possibilities

The second factor of the SOAR framework is Opportunities. From the early study on strategic issue diagnosis and labeling in organizations (Dutton et al., 1983), Fredrickson (1985) operationalized opportunities as situations in which gains could be made, and Jackson and Dutton (1988) defined opportunities as situations or issues with a high likelihood of positive success. The strategic and future-oriented attributes of opportunities is also seen in the field of entrepreneurship in which opportunities involve the processes of discovery and evaluation in individuals leading to the creation of new outcomes (Shane and Venkataraman, 2000; Eckhardt and Shane, 2003). In the field of POS, opportunities refer to individuals working together to positively affect the future (Bushe, 2007). The Opportunities factor in SOAR is defined as those situations, ideas, or possibilities that make it possible to turn visions into reality.

Cameron and Lavine (2006) explored the extraordinary success achieved by focusing on opportunities during the clean-up and the closure of the Rocky Flats nuclear weapon production facility, which was termed by the media as the most dangerous building in the United States. Their abundance approach strives for positive deviance in pursuing the best of the human condition and working to fulfill the highest potential of organizations and individuals through a pursuit of opportunities, ideas, and possibilities. This brings opportunities into the realm of ideas and possibility thinking about a desired future through shared dialogues about opportunities as new ideas and possibilities within an innovative and adventurous activity of exchange (Stavros and Torres, 2018). Applying an abundance approach helps tap into strengths and then identify ideas and innovations that leverage these strengths, leading to more generative ways of strategizing. As such, the application of abundance theory offers a generative approach for identifying opportunities to bridge the gap between current performance and potentiality. Like the abundance approach, SOAR focuses on opportunities, ideas, and possibilities in an organization.

Opportunities in organizations have been traditionally measured by the SWOT analysis as an analytical tool or a checklist, usually completed by individuals about the organization (Helms and Nixon, 2010; Puyt et al., 2020). Ames and Runco (2005) interviewed entrepreneurs about their self-reported opportunities using the SWOT model. Scheaf et al. (2020) developed a 14-item self-report measure of opportunity evaluation in which entrepreneurial opportunities are rated in three domains: gain estimation (e.g., I see large potential gains for myself in pursuing the opportunity), perceived feasibility (e.g., I have what it takes to create opportunities), and loss estimation (e.g., For me, the potential for loss in pursuing the opportunity is high). Davidsson et al. (2021) developed the four-item Venture Idea Assessment to measure self-reported ratings of an entrepreneurial idea. For example, “How confident are you that a person with the right knowledge and motivation should be encouraged to act on this idea.”

The SOAR Scale combines self-reported identification of and use of opportunities, ideas, and possibilities in one tool *via* three items that measure the first-order factor, Opportunities, along a five-point rating scale (“never” to “always”). Each item is preceded by the phrase “When you think strategically, how often do you focus on…” Opportunities (those situations that make it possible to turn personal, team, or organizational visions into reality), Ideas (thoughts or suggestions for possible courses of action to positively change the future), and Possibilities (those innovative conditions that may lead to personal, team, or organizational success).

#### Aspirations Factor: Aspirations, Wishes, Desires

The third factor of the SOAR framework is Aspirations. From the root aspire, or steadfast intention for a higher goal, the concept of aspirations was defined in the field of psychology by Haller (1968) as “the cognitive orientational aspect of goal-direct behavior” (p. 484). Self-determination theory considers aspirations (e.g., goals, affiliations, personal growth, and community contribution) as a basic need satisfaction enhancing wellbeing (Deci and Ryan, 1985, 2000). In the context of strategy, aspirations represent the vision and support of a long-term strategy that drives operational strategy (Mintzberg, 1973; Steiner, 1979). Porter (1980) noted aspirations are realized through the incorporation of organizational strengths and capabilities. Aspirations are closely linked with the concepts of wishes and desires (Hart, 2016). For example, aspirations are linked with wishes when describing goals that are most important for individuals and organizations. Aspirations are linked with desires when describing aspirations as a self-expressed view of the future based on goal-attainment and decision-making (Perugini and Bagozzi, 2004; Boccagni, 2017). The aspirations factor in SOAR is defined as the dreams, vision, wishes, or desires to achieve personal, team, or organizational goals and objectives.

Aspirations expand and give voice to the wishes and desires of those focusing on what stakeholders care deeply about in the organization. Frequently strategic management research of aspirations is embedded in the discourse on visioning in organizations. This aspect of strategic management is described as symbolic and viewed as interpretive, being expressed as metaphors and frames of reference that enable the organization and its environment to understand stakeholder aspirations, wishes, and desires (Chaffee, 1985; Hart, 1992). In some instances, visioning clarifies aspirations by helping organizational members confront uncertainty and resolve confusion through visual images and verbal expressions of where they want to go (Bolman and Deal, 2008). The visioning mode entails cognitively constructing conceptual representation of the aspirations that will guide future actions (Strange and Mumford, 2005). Research has validated the positive impact of visioning on both employee productivity and organizational-level performance as measured in growth and profitability (Baum et al., 1998).

A SOAR-based view considers aspirations as playing a significant role in a positive view of strategy. Aspirations describe the organization’s strategic intent by taking into account its strengths and opportunities and then explicitly stating what one wishes or desires for the benefit of stakeholders (Stavros and Hinrichs, 2009). Aspirations represent how to convey an envisioned future and the roadmap for creating this future. The supporting pillar of an organization’s aspirations is its core ideology that defines its character through a consistent identity that transcends changes in leadership, market life cycles, and technological breakthroughs (Collins and Porras, 1996). Aspirations reflect an organization’s future directions or compelling dreams. This in turn energizes stakeholders and provide the emotional and intellectual energy for a collaborative journey into the future (Hamel et al., 1989; Prahalad and Hamel, 1994).

Strategic intent describes the organization’s mission and strategy to reach its vision. Beneficial change results from the strategic intention, vision, and needs of an organization (Prahalad and Hamel, 1994). Strategic intent challenges the organization to create an alignment among strengths, opportunities, and aspirations (Hamel and Prahalad, 2005). Therefore, the emphasis on strategic intent is to leverage current strengths and opportunities to acquire new resources to accomplish aspirations. SOAR also acknowledges the value of visioning as a tool to inspire and guide action based on others’ aspirations. The framework invites participants to visualize the organization’s future by asking questions designed to elicit a vision of its desired future and outcomes through a discussion of individuals’ aspirations, wishes, and desires.

Aspirations, in the context of psychology, have been measured by the Aspiration Index (Kasser and Ryan, 1993, 2001), a 35-item self-report measure of aspirational items (e.g., “you will be the one in charge of your life”).

The SOAR Scale combines self-reported identification of and use of aspirations, values, and desires in one tool *via* three items that measure the first-order factor, Aspirations, along a five-point rating scale (“never” to “always”). Each item is preceded by the phrase “When you think strategically, how often do you focus on…” Aspirations (dream or vision to achieve personal, team, or organizational goals and objectives), Wishes (a hope for achieving personal, team, or organizational goals and objectives), and Desires (your intention for personal, team, or organizational success).

#### Results Factor: Results, Completed Tasks, Outcomes

The fourth factor of the SOAR framework is Results. According to the online etymology dictionary, the noun results is from the 17th century and refers to consequences, effects, and outcomes^2^. In the field of psychology, results imply goal-attainment, task attainment (Locke et al., 1968; Locke and Latham, 1990), and outcomes (Salmoni et al., 1984). The strategic focus of a results-oriented work environment is to obtain goals and outcomes and to complete tasks (Santalainen and Hunt, 1988; Thierry, 1998; Binnendijk, 2001; Burke, 2002; Ressler and Thompson, 2008; Verbeeten and Speklé, 2015). From a POS perspective, a results-oriented strategy embraces a broaden-and-build perspective by framing the attainment of performance outcomes as opportunities for learning, growth, and expanded awareness (Cravens et al., 2010). The results factor in SOAR is defined as the measured goals, completed tasks, and outcomes obtained for success.

The results emphasis of SOAR holds organizational members accountable for completing tasks and achieving outcomes. Managers have been attempting to move accountability inside the activity of working for decades instead of locating it after something or someone has gone wrong and asking him or her to explain and rationalize. This constructive accountability recognizes and emphasizes that the performance of an organization’s member impacts the performance of others. Thus, accountability is experienced and understood as constructive and contributing to mutual accomplishment during the completion of work tasks (Seiling and Hinrichs, 2005). It occurs spontaneously and purposely during the exchange of conversations that include suggestions, acknowledgments, answers, challenges of right or wrong, and opportunities to contribute to the success of others. Inside these task-related activities, there is an exchange of accountability that is constructive—people purposely join to be responsible and accomplish the work based on the desired outcomes.

Strengths, Opportunities, Aspirations, and Results activates constructive accountability, exponentially expanding energy and interest in contributing to the welfare within the organization. For example, John Deere has been using SOAR at multiple levels for seven years with great success. At Deere, there were also lagging indicators including: “a 50% reduction in project completion cycle time; a 25% increase in stretch projects and goals; and accomplishments of SBU and functional department goals” (Hinrichs, 2010, p. 34). People pull themselves and others into the process to create a future that is mutually beneficial. Commitment and dedication to the future spread across the group and extend outward to other groups. They become constructively accountable for deciding and performing what tasks must be done to reach results. This requires perspective taking and reframing so the organization can envision new ways to approach its strengths, opportunities, and aspirations (Beatty and Hughes, 2005). Reframing is an important aspect of visioning because it challenges organizational members to reflect upon what they can do differently and to follow up these reflections with system thinking that maps out the interrelationships of variables to produce extraordinary results (Senge, 1990; Collins, 2001).

While aspirations motivate and energize organizational members to act in ways consistent with producing results for the organization (Hart, 1992), how does one know when it is succeeding at producing these results? From a positive strategy perspective, this is a conscientious identification and use of meaningful measures that indicate whether everything is on track to achieve its goals considering its strengths, opportunities, and aspirations (Stavros and Hinrichs, 2009). Two prominent approaches to measuring results in the field of strategy are the balanced scorecard (BSC) and the triple bottom line (TBL).

The BSC was designed as a management and measurement system for specifying organizational strategies and translation of strategic vision into action items and outcomes (Kaplan and Norton, 2006). The BSC provides a lens to explore how results can be enacted through objectives and complete tasks to achieve desired outcomes. The BSC tells the story of an organization’s strategy through a results-oriented measurement system indicating the present and future prospects of an organization (Huang, 2009). Conceptualizing the results dimension of the SOAR framework from a BSC view illustrates the importance of linking results to strengths, opportunities, and aspirations by providing a map for how organizations can convert assets and capabilities into strategic initiatives (tasks) and desired outcomes (Kaplan and Norton, 2006). A key point regarding the measurement system is the focus on more meaningful results than solely profit. Overtime, the BSC has been extended to include social and environmental measurement (Hubbard, 2009). In some instances, these additional components are integrated from a TBL perspective (Jamali, 2006), which offers another lens for people in organizations to incorporate the results aspects of SOAR. TBL emphasizes that, when results are measured at the organizational level, there are multiple impacts on society with associated bottom lines. TBL is a value-based aspiration that requires the organization to acknowledge the relationship between its economic performance and its performance in social and environmental terms (Colbert and Kurucz, 2007; Wirtenberg et al., 2017). TBL now is being used to capture the values, visions (aspirations), opportunities, and practices that organizations use to maximize positive impact and generate sustainable value for the economy, the society, and the environment (Elkington, 1998; Jamali, 2006; Laszlo and Cooperrider, 2010).

In addition to the BSC and the TBL, rapid tools such as the Job Aid Eight-Point Checklist for Mission and Vision Statement (Moore et al., 2011) have been developed to quickly assess results-oriented mission and vision statement for the organization [e.g., “Are all objectives linked to (and aligned with) results and consequences for individuals and small groups (Micro), the organization (Macro), or external clients and society (Mega)?”]. The SOAR Scale measures the first-order factor, Results, along a five-point rating scale (“never” to “always”) using three items. Each item is preceded by the phrase “When you think strategically, how often do you focus on…” Results (the measured goals obtained for success), Completed Tasks (activities that when completed help achievement of desired results), and Outcomes (those meaningful achievements that will have a positive result).

## The Current Study

Demonstrating the reliability and validity of the SOAR Scale is necessary if researchers are to have confidence reporting the results obtained from it. Additionally, researchers should know if the SOAR Scale can be used to generate a substantively meaningful total score, or if the four subscale scores should be used for interpretations. Accordingly, the purpose of this study was to assess the measurement properties of the SOAR Scale in a sample of U.S. working professionals. We assessed the scale’s reliability, construct validity, dimensionality, and measurement invariance by gender, age, and education.

## Research Methods

### Study Sample

Participants (*N* = 308) were recruited from invitations posted in the following LinkedIn groups to complete an online survey *via* the online survey tool SurveyMonkey: AI, leadership, strategic planning, change management, project management, financial management, and general business management. The inclusion criterion was a working professional in the U.S. The project was approved by the Lawrence Technological University Institutional Review Board (IRB). Due to incomplete data, *n* = 23 participants were excluded from the current study, resulting in an analytical sample size of *N* = 285. Participants were comprised of professionals from a variety of industries (automotive, consulting, education, engineering, finance, government, healthcare, IR, and marketing) and positions (administrative, executive, director, manager, supervisor, and VP). The sample was relatively matched by gender (female = 53%, male = 47%) but was not matched by age (16.5% 18–34 years of age, 47.0% 35–54 years of age, 36.5% 55–74 years of age) or level of education (undergraduate degree = 23.5%, graduate degree = 76.5%).

### Instrument

The 12-item SOAR Scale is a self-report survey on SOAR-based capacity. This survey assesses an individual’s natural capacity for strategic thinking, planning, conversations, and leading from a SOAR-based, generative perspective. The SOAR Scale was created for members of any organization. The survey is organized into four first-order factors representing the four elements of SOAR. Items were selected to support our *a priori* assumptions about the SOAR framework theory and hypothesized patterns of item loadings onto their respective factors. Each first-order factor is measured by three items scored along a five-point rating scale (“never” to “always,” 1–5), with each item preceded by the phrase “When you think strategically, how often do you focus on…”. Users should organize the items alphabetically and include the corresponding definition.

The first-order factor Strengths is measured by Strengths (those natural capacities in self and others that allow optimal performance), Assets (those competencies that create personal, team, or organizational values), and Capabilities (those abilities that create the best for yourself, your team, or organization). The first-order factor Opportunities is measured by Opportunities (those situations that make it possible to turn personal, team, or organizational visions into reality), Ideas (thoughts or suggestions for possible courses of action to positively change the future), and Possibilities (those innovative conditions that may lead to personal, team, or organizational success). The first-order factor Aspirations is measured by Aspirations (dreams or visions to achieve personal, team, or organizational goals and objectives), Wishes (a hope for achieving personal, team, or organizational goals and objectives), and Desires (your intention for personal, team, or organizational success). The first-order factor Results is measured by Results (the measured goals obtained for success), Completed Tasks (activities that when completed help in the achievement of the desired results), and Outcomes (those meaningful achievements that will have a positive result).

### Statistical Analyses

The survey data were analyzed using SPSS v28 and Mplus v8.6. First, we started with exploring the factorial structure of the SOAR Scale using exploratory factor analysis (EFA). Kaiser–Meyer–Olkin (KMO) > 0.60 and Bartlett’s sphericity test chi-square < 0.01 were first determined as support for factorability (Kaiser, 1974), followed by using Mplus to conduct EFA based on maximum likelihood estimation (ML) and Geomin Oblique rotation. Mplus was also used to conduct Bifactor EFA as an alternative to EFA in which ML estimation and Bi-Geomin Orthogonal rotation was used to explore if a general factor influences all items and if-specific factors, uncorrelated with the general factor, influence sets of items (Muthén and Asparouhov, 2016). Given our *a priori* hypothesized structure of the data measuring four factors, EFA was specified to extract a fixed number of four factors (i.e., Strengths, Opportunities, Aspirations, and Results), and Bifactor EFA was specified to extract a fixed number of five factors (i.e., General Factor, Strengths, Opportunities, Aspirations, Results; Hurley et al., 1997; Watkins, 2018), with Eigenvalues > 1, item loadings > 0.40, and overall percent of variance ≥ 50 (Streiner, 1994; Muthén and Muthén, 2017).

Second, a competing measurement modeling strategy was employed in Mplus using the confirmatory factor analytical (CFA) and the exploratory structural equation modeling (ESEM; Asparouhov and Muthén, 2009) to investigate the construct validity of the SOAR Scale. The models were estimated using the robust maximum likelihood (MLR) estimator which addresses slight or moderate assumptions in normality of the SOAR Scale 5-point Likert rating scale data (Li, 2016; Tóth-Király et al., 2018). For CFA models, all items were estimated to load onto their *a priori* theoretical factors, cross-loadings were constrained to zero, and errors terms were allowed to correlate (Wang and Wang, 2020). ESEM measurement models were specified and estimated in Mplus using guidelines outlined in the study of Van Zyl and Ten Klooster (2022). The Mplus syntax was created using an ESEM online code generator created by De Beer and Van Zyl (2019).

(0) Model 0, the Unidimensional CFA Model of Overall SOAR (established a baseline model from which other models were compared).
(1) Model 1, the Independent Cluster Model CFA (ICM CFA) Model comprised of Strengths, Opportunities, Aspirations, and Results (an estimated model in which the four first-order factors of the SOAR Scale were correlated).
(2) Model 2, the Hierarchical CFA (H CFA) Model compromises a single second-order factor of SOAR, consisting of four first-order factors (estimated the hierarchical nature of the SOAR Scale in which the four first-order factors load onto a second-order general SOAR factor).
(3) Model 3, the Bifactor CFA Model of Overall SOAR (estimated the dimensionality of the SOAR Scale *via* Orthogonal Target rotation and constraining to zero the relationships between specific and general factors to determine if the SOAR Scale is unidimensional or multidimensional; cf. Reise, 2012).
(4) Model 4, the ESEM Model comprised of SOAR Scale items that estimated to load onto their four *a priori* theoretical factors and cross-loadings were permitted but targeted to be close to zero).
(5) Model 5, the Hierarchical ESEM (H ESEM) Model compromises a single second-order factor of SOAR, consisting of four first-order factors (the SOAR Scale items were estimated to load onto their four *a priori* theoretical factors and cross-loadings were permitted but targeted to be close to zero; then, an ESEM-within-CFA estimation procedure was used to construct the higher-order factorial model in which the starting values for items were constrained to the unstandardized factor and cross-loadings from Model 4).
(6) Model 6, the Bifactor ESEM Model of Overall SOAR (similar to Model 3, but cross-loadings were permitted but targeted to be close to zero).

These models were evaluated first for the acceptable model fit according to the following criteria: χ^2^/*df* < 3, CFI > 0.90, TLI > 0.90, RMSEA < 0.08, SRMR < 0.08 (Chen, 2007; Wang and Wang, 2020). Next, the measurement quality among the models with acceptable fit was determined by a thorough examination of parameter estimates to determine the best fitting model (Morin et al., 2016, 2020; Tóth-Király et al., 2020; Van Zyl and Ten Klooster, 2022). Specifically, we examined standardized factor loadings (e.g., significant loadings with λ > 0.35 and expected pattern of loadings), item uniqueness (e.g., residual error variances δ > 0.10 but < 0.90), cross-loadings (e.g., λ < 0.5), and factor correlations (e.g., the model with the smallest factorial intercorrelations between the latent factors may be the best fitting model).

Descriptive statistics were comprised of item means (x̄), standard deviations (σ), Skewness, Kurtosis, and corrected item-total correlations (CITC). The criteria for normality of the data were Skewness and Kurtosis < 2 (Kim, 2013), and the criterion for item representation of corresponding factor was CITC > 0.30 (Zijlmans et al., 2019). Item level reliability was determined by the Cronbach’s coefficient alpha (α) > 0.70 (Nunnally and Bernstein, 1994), whereas factor level reliability was determined by McDonald’s omega coefficient of composite reliability (ω) > 0.70 (Morin et al., 2020) and average variance extracted (AVE) > 0.50 (Kline, 2015).

Finally, to determine whether the results of this study could be assumed to generalize across studies, the Mplus was used to assess measurement invariance of the optimal model across gender, age, and education (Rudnev et al., 2018, p. 48) using a series of increasingly restrictive levels of measurement invariance in terms of configural, metric, scalar, and strict invariance (Millsap, 2012). Following the procedure for testing invariance across groups described by Rudnev et al. (2018), we estimated six invariance models:

Model 1: Configural invariance (general factor structure is the same across groups).
Model 2: Metric invariance (first-order factor loadings are the same across groups).
Model 3: Scalar invariance (first-order factor loadings and observed item intercepts are the same across groups).
Model 4: Strict invariance (factor loadings, observed item intercepts, and residual errors are the same across groups).
Model 5: Factor variance and factor covariance invariance (factor variance and factor covariance are the same across groups).
Model 6: Factor mean invariance (factor means are the same across groups).

Evidence for measurement invariance was established as two-fold: First, the model fit of each invariance model was evaluated using the study criteria of χ^2^/*df* < 3, CFI > 0.90, TLI > 0.90, RMSEA < 0.08, and SRMR < 0.08. Second, each ever-restrictive invariance model was compared by the robust (Satorra–Bentler) chi-square different test (Δχ^2^) calculated from loglikelihood (Satorra and Bentler, 2010), and it compared the absolute change in the CFI, RMSEA, and SRMR between invariance models. We considered the following when assessing invariance: Δχ^2^ *p* > 0.05, ΔCFI ≤ 0.010, ΔRMSEA ≤ 0.015, and ΔSRMR ≤ 0.03 (Chen, 2007; Fisher et al., 2020; Wang and Wang, 2020).

## Results

### Exploratory Factor Analysis

An analysis of the SOAR Scale started with exploring its factorial structure in the study sample using the EFA. Results for KMO measure of sampling adequacy (KMO = 0.846) and Bartlett’s test of sphericity (significant at *p* < 0.001) support the extraction of meaningful factors in EFA. Given our *a priori* hypothesized structure of the data measuring four factors, EFA was specified to extract four factors (the Bifactor EFA was specified to extract five factors, i.e., the four *a priori* specific factors and one general factor). Model fit EFA: χ^2^ = 52.43, df = 24, *p* = 0.001; RMSEA = 0.064 [0.041,0.089]; CFI = 0.979; TLI = 0.942; SRMR = 0.23; AIC = 5922.67; BIC = 6163.74; aBIC = 5954.45. Model fit Bifactor EFA: χ2 = 25.00, df = 16, *p* = 0.07; RMSEA = 0.044 [0.000,0.076]; CFI = 0.993; TLI = 0.972; SRMR = 0.013; AIC = 5911.24; BIC = 6181.52; aBIC = 5946.87.

The left panel of Table 1 shows the item loadings and the percentage of variance for the four-factor EFA model, and the right panel of Table 1 shows the item loadings for the bifactor EFA model. Items that significantly loaded onto their respective *a priori* factors are shown in bold font (*p* < 0.05). EFA results found the four factors that had Eigenvalues > 1 and that accounted for 71.9% of the overall variance. Our *a priori* factorial structure of the Strengths (λ = 0.59–0.73), Opportunities (λ = 0.56–0.79), Aspirations (λ = 0.66–0.87), and Results (λ = 0.58–0.85) factors was supported. In contrast to the EFA, the Bifactor EFA results found the 12 SOAR Scale items significantly loaded onto a general factor (λ_1_ = 0.30–0.72) but did not maintain the expected pattern of loadings in the four specific factors (i.e., λ_2_ to λ_5_). These results suggest that the EFA supported our *a priori* assumptions about the loadings of the 12 SOAR Scale items onto the Strengths, Opportunities, Aspirations, and Results factors. We next proceeded to analyze the factorial structure of the SOAR Scale using CFA and ESEM.

**TABLE 1:** Exploratory factor analysis and bifactor EFA of the SOAR Scale.

	EFA				Bifactor EFA				
Item	λ _1_	λ _2_	λ _3_	λ _4_	λ _1_	λ _2_	λ _3_	λ _4_	λ _5_
S1. Strengths	**0.591**	0.183	0.101	–0.022	**0.678**	0.037	0.263	–0.010	–0.030
S2. Assets	**0.731**	–0.054	0.062	0.045	**0.610**	–0.040	**0.470**	–0.003	0.019
S3. Capabilities	**0.685**	0.214	–0.107	0.010	**0.671**	–0.037	0.289	–0.033	0.039
O1. Opportunities	0.084	**0.669**	0.016	0.025	**0.694**	–0.038	0.281	–0.024	0.001
O2. Ideas	0.045	**0.558**	0.002	0.091	**0.570**	0.028	–0.229	–0.018	0.085
O3. Possibilities	–0.015	**0.789**	0.059	0.004	**0.715**	0.004	**–0.368**	0.022	–0.001
A1. Aspirations	0.002	–0.018	**0.866**	0.000	**0.459**	0.187	0.002	0.285	–0.116
A2. Wishes	–0.013	0.009	**0.813**	0.026	**0.469**	0.128	–0.001	1.620	–0.014
A3. Desires	0.039	0.144	**0.660**	–0.012	**0.533**	1.947	–0.005	0.062	–0.019
R1. Results	0.070	0.006	0.017	**0.765**	**0.476**	–0.024	0.051	–0.057	**0.636**
R2. Completed Tasks	0.169	–0.162	0.008	**0.580**	**0.301**	–0.050	**0.189**	0.015	**0.493**
R3. Outcomes	–0.097	0.121	–0.012	**0.839**	**0.460**	–0.042	–0.099	–0.045	**0.722**
R^2^	38.4%	15.4%	9.4%	8.7%					

*λ = Factor loadings. EFA statistics based on maximum likelihood estimation (ML) and Geomin Oblique rotation; Bifactor EFA statistics based on ML estimation and Bi-Geomin Orthogonal rotation. Bold factor loadings are significant at p < 0.05.*

### Measurement Models

Table 2 presents the goodness-of-fit indexes and the information criteria associated with the competing CFA and ESEM measurement models. The Unidimensional CFA solution (Model 0) did not fit the data well (χ^2^/*df* > 3, CFI and TLI < 0.90, RMSEA and SRMR > 0.08). In contrast, the ICM CFA (Model 1), the H CFA (Model 2), the Bifactor CFA (Model 3), the ESEM (Model 4), the H ESEM (Model 5), and the Bifactor ESEM (Model 6) solutions provided an acceptable degree of fit to the data (χ^2^/*df* < 3, CFI and TLI > 0.90, RMSEA and SRMR < 0.08). In comparing the three competing CFA measurement models, the ICM CFA solution had better fit than the H CFA and Bifactor CFA solutions (CFI = 0.967 vs. 0.959,0.961; TLI = 0.955 vs. 0.946,0.939; RMSEA = 0.051 vs. 0.056,0.060; SRMR = 0.039 vs. 0.051, 0.047). In comparing the three ESEM measurement models, the ESEM solution had a better fit than the H ESEM solution (CFI = 0.977 vs. 0.971; TLI = 0.937 vs. 0.926; RMSEA = 0.060 vs. 0.065; SRMR = 0.019 vs. 0.027). However, the Bifactor ESEM solution had a slightly better fit than the ESEM solution (CFI = 0.986 vs. 0.977; TLI 0.942 vs. 0.937; RMSEA = 0.058 vs. 0.060; SRMR = 0.016 vs. 0.019). These results suggest that the first-order solutions had better fit than the hierarchical solutions. The results also suggest that the Bifactor ESEM solution may have better fit than the ESEM solution. To help with selecting the best fitting final model, we also examined the measurement quality among the ICM CFA, ESEM, and Bifactor ESEM models.

**TABLE 2:** Goodness-of-fit statistics for the models estimated on the SOAR Scale.

Model	Description	χ ^2^	*df*	χ ^2^/*df*	CFI	TLI	RMSEA	SRMR	AIC	BIC	aBIC	Meets Criteria
Model 0	Unidimensional CFA	496.98	54	9.20	0.595	0.505	0.170 [0.156,0.183]	0.113	6389.14	6520.63	6406.47	No
Model 1	ICM CFA	83.67	48	1.74	0.967	0.955	0.051 [0.032,0.069]	0.039	5918.80	6072.20	5939.02	Yes
Model 2	H CFA	94.85	50	1.90	0.959	0.946	0.056 [0.039,0.073]	0.051	5928.39	6074.49	5947.65	Yes
Model 3	Bifactor CFA	84.39	42	2.01	0.961	0.939	0.060 [0.041,0.078]	0.047	5929.78	6105.10	5952.89	Yes
Model 4	ESEM	49.02	24	2.04	0.977	0.937	0.060 [0.036,0.085]	0.019	5922.67	6163.74	5954.45	Yes
Model 5	H ESEM	57.74	26	2.22	0.971	0.926	0.065 [0.043,0.088]	0.027	5926.92	6160.68	5957.74	Yes
Model 6	Bifactor ESEM	31.46	16	1.97	0.986	0.942	0.058 [0.027,0.088]	0.016	5911.24	6181.52	5946.87	Yes

*Statistics based on maximum likelihood estimation (ML), χ^2^, Chi-square; df, degrees of freedom; CFI, Comparative Fit Index; TLI, Tucker–Lewis Index; RMSEA, Root Mean Square Error of Approximation [90%CI]; SRMR, Standardized Root Mean Square Residual; AIC, Akaike Information Criterion; BIC, Bayes Information Criterion; aBIC, Adjusted Bayes Information Criterion. The goodness-of-fit criteria for acceptable model fit: χ^2^/df < 3, CFI > 0.90, TLI > 0.90, RMSEA < 0.08, SRMR < 0.08 (Chen, 2007; Wang and Wang, 2020).*

### Independent Cluster Model Confirmatory Factor Analytical vs. Exploratory Structure Equation Modeling

Before examining the Bifactor ESEM solution, we first examined the measurement quality among the ICM CFA and ESEM solutions through the inspection of the standardized parameter estimates (see Table 3). Results found well-defined CFA and ESEM factors with significant λ > 0.35 and expected patterns of loadings, item uniqueness was acceptable (δ > 0.10 but < 0.90), and all cross-loadings in the ESEM solution were small (λ < 0.20). Model-based omega coefficients of composite reliability found that the Strengths, Opportunities, and Aspirations factors were slightly higher for the CFA solution vs. the ESEM solution (ω = 0.777,0.768, and 0.848 vs. 0.753,0.701, and 0.839). Omega for the Results factor was slightly higher for the ESEM solution vs. the CFA solution (ω = 0.791 vs. 0.789).

**TABLE 3:** Standardized parameter estimates from the ICM CFA (Model 1) and ESEM Solutions (Model 4).

						CFA		ESEM				
Factor/Item	$\bar{x}$	σ	Skew	Kurt	CITC	Factor (λ)	δ	STR (λ)	OPP (λ)	ASP (λ)	RES (λ)	δ
**Strengths (STR) α = 0.773, AVE = 0.538**												
Strengths	4.18	0.62	–0.41	0.71	0.60	0.745**	0.446	0.628**	0.113	0.098	–0.026	0.464
Assets	3.78	0.70	–0.58	0.54	0.51	0.705**	0.502	0.768**	–0.135*	0.055	0.043	0.431
Capabilities	4.12	0.57	–0.22	1.06	0.55	0.750**	0.437	0.729**	0.139	–0.114*	0.008	0.418
ω						0.777		0.753				
**Opportunities (OPP) α = 0.765, AVE = 0.527**												
Opportunities	4.09	0.63	–0.58	0.37	0.56	0.753**	0.433	0.102	0.651**	0.021	0.022	0.468
Ideas	4.25	0.59	–0.33	0.59	0.51	0.636**	0.595	0.056	0.547**	0.006	0.090	0.605
Possibilities	4.11	0.62	–0.53	0.40	0.58	0.780**	0.391	0.001	0.779**	0.067	0.000	0.338
ω						0.768			0.701			
**Aspirations (ASP) α = 0.842, AVE = 0.650**												
Aspirations	3.68	0.78	–0.58	0.05	0.52	0.845**	0.286	–0.004	–0.044	0.881**	–0.003	0.263
Wishes	3.99	0.90	–0.70	–0.17	0.54	0.816**	0.335	–0.019	–0.014	0.827**	0.023	0.334
Desires	3.59	0.83	–0.66	0.10	0.53	0.755**	0.431	0.041	0.118	0.671**	–0.016	0.434
ω						0.848				0.839		
**Results (RES) α = 0.765, AVE = 0.561**												
Results	4.16	0.66	–0.63	1.06	0.51	0.829**	0.313	0.039	0.003	0.014	0.780**	0.353
Completed Tasks	3.86	0.82	–0.70	0.27	0.34	0.568**	0.678	0.148	–0.175**	0.004	0.592**	0.631
Outcomes	4.14	0.65	–0.54	0.93	0.48	0.821**	0.326	–0.138*	0.137*	–0.013	0.855**	0.265
ω						0.789					0.791	

$\bar{x}$, mean; σ, standard deviation; Skew, skewness; Kurt, kurtosis; CICT, corrected item-total correlation; α, item-based Cronbach’s alpha reliability; AVE, average value explained; ω, model-based omega composite reliability; λ, standardized factor loadings; δ, item uniqueness/residual. Target factor loadings are in bold. *p < 0.05, **p < 0.01.

Next, we examined the standardized latent factor correlations between the ICM CFA and the ESEM solutions (see Table 4). Results found that factor correlations were lower for ESEM (*r* = 0.184 to 0.505, Mean = 0.428) than ICM CFA (*r* = 0.201 to 0.627, Mean = 0.483). These results suggest the ESEM solution that provides a clearer differentiation between the four SOAR factors than ICM CFA and it should therefore be considered as the optimal model (Van Zyl and Ten Klooster, 2022). Since the Bifactor ESEM had a slightly better model than ESEM, we needed to thoroughly examine the Bifactor ESEM solution before determining the best fitting final model.

**TABLE 4:** Standardized latent factor correlations from the ICM CFA (Model 1) and ESEM solutions (Model 4).

	STR	OPP	ASP	RES
Strengths (STR)	–	0.505**	0.453**	0.498**
Opportunities (OPP)	0.627**	–	0.471**	0.462**
Aspirations (ASP)	0.489**	0.530**	–	0.184*
Results (RES)	0.517**	0.534**	0.201*	–

CFA (Model 1) correlations are under the diagonal; ESEM (Model 4) correlations are above the diagonal. *p < 0.05, **p < 0.01.

### Exploratory Structure Equation Modeling vs. Bifactor Exploratory Structure Equation Modeling

The standardized parameter estimates from the Bifactor ESEM are reported in Table 5. The Bifactor ESEM solution shows that a general factor is not well-defined since there were no significant target loadings from the SOAR Scale items onto the G factor (λ = 0.141 to 1.407, *p >* 0.05). Furthermore, the expected pattern of loadings onto the four specific factors did not occur. Finally, item uniqueness was not acceptable in this solution (δ = –3.08 to 0.66). Taken together, these results do not support Bifactor ESEM as an acceptable solution. Thus, ESEM was selected as the best fitting final model.

**TABLE 5:** Standardized parameter estimates from the bifactor ESEM solution (Model 6).

Factor/Item	G Factor (λ)	STR (λ)	OPP (λ)	ASP (λ)	RES (λ)	δ
Strengths (STR)						
Strengths	**0.340**	**0.585**	0.235	0.036	0.129	0.469
Assets	**0.272**	0.698*	0.055	–0.029	0.173	0.405
Capabilities	**0.282**	0.607*	0.222	–0.008	0.204	0.461
ω		0.728				
Opportunities (OPP)						
Opportunities	**0.295**	0.215	0.636*	–0.013	0.157	0.437
Ideas	**0.278**	0.148	0.493*	0.035	0.204	0.614
Possibilities	**0.360**	0.145	**0.689**	–0.009	0.148	0.353
ω			0.702			
Aspirations (ASP)						
Aspirations	**0.515**	0.186	0.190	**–0.030**	–0.059	0.660
Wishes	**1.407**	–0.150	–0.150	**–0.910**	–0.113	–1.865
Desires	**1.361**	–0.090	–0.086	**1.485**	–0.084	–3.081
ω				–0.074		
Results (RES)						
Results	**0.192**	0.202	0.165	0.016	0.730**	0.361
Completed Tasks	**0.141**	0.216	–0.027	–0.050	0.550**	0.628
Outcomes	**0.185**	0.067	0.244	–0.006	0.805**	0.254
ω	1.01				0.778	

ω, model-based omega composite reliability; λ, standardized factor loadings; δ, item uniqueness/residual. Target factor loadings are in bold. *p < 0.05, **p < 0.01.

### Measurement Invariance

Finally, given that the ESEM solution (Model 4) was the optimal model, we estimated the measurement and structural invariance of the SOAR Scale across gender, age, and education groups with the aid of an online ESEM invariance syntax generator for Mplus (De Beer and Morin, 2022).

#### Invariance Across Gender Groups

The top section of Table 6 presents the results of the measurement invariance tests of the SOAR Scale across gender (women vs. men) in the ESEM model. Results found evidence for configural, metric, and scalar invariance but not strict invariance. Accordingly, no additional invariance tests were conducted. However, in an attempt to compare latent means on the SOAR Scale factors, mean scores in women were constrained to zero within the strict invariance model, and Strengths, Opportunities, Aspirations, and Results in men were freely estimated. Latent means between men and women across all factors were *p* > 0.05, finding no difference between men and women in latent means.

**TABLE 6:** Measurement invariance for the final retained model (four-factor first-order ESEM, Model 4).

Model	χ ^2^	*df*	CFI	RMSEA	SRMR	Model Comparison	Δ χ ^2^	*df*	*p*	Δ CFI	Δ RMSEA	Δ SRMR	Invariance
**Gender Groups**													
G1. Configural	81.24	48	0.971	0.070	0.023								
G2. Metric	99.55	80	0.983	0.041	0.046	G2 vs. G1	22.631	32	0.890	0.012	–0.029	0.023	Yes
G3. Scalar	104.56	88	0.985	0.036	0.050	G3 vs. G2	4.709	8	0.788	0.002	–0.005	0.004	Yes
G4. Strict	132.80	100	0.971	0.048	0.069	G4 vs. G3	26.717	12	0.008	–0.014	0.012	0.019	No
**Age Groups**													
A1. Configural	121.57	72	0.959	0.085	0.026								
A2. Metric	179.21	136	0.964	0.058	0.086	A2 vs. A1	67.392	64	0.362	0.005	–0.027	0.060	No
A3. Scalar	211.90	152	0.950	0.064	0.085	A3 vs. A2	33.716	16	0.006	–0.014	0.006	–0.001	No
A4. Strict	245.02	176	0.943	0.064	0.164	A4 vs. A3	33.169	24	0.101	–0.007	0.000	0.079	No
**Education Groups**													
E1. Configural	110.91	48	0.947	0.096	0.027								
E2. Metric	125.53	80	0.961	0.063	0.064	E2 vs. E1	32.659	32	0.434	0.014	–0.033	0.037	Yes
E3. Scalar	137.69	88	0.958	0.063	0.065	E3 vs. E2	12.089	8	0.147	–0.003	0.000	0.001	Yes
E4. Strict	147.48	100	0.960	0.058	0.093	E4 vs. E3	11.079	12	0.522	0.002	–0.005	0.028	Yes
E5. Factor Variance-Covariance	158.05	110	0.959	0.055	0.135	E5 vs. E4	11.412	10	0.326	–0.001	–0.003	0.042	Yes
E6. Factor Means	163.18	114	0.958	0.055	0.131	E6 vs. E5	5.128	4	0.274	–0.001	0.000	–0.004	Yes

*χ^2^, chi-square; df, degrees of freedom; CFI, Comparative Fit Index; RMSEA, Root Mean Square Error of Approximation; SRMR, Standardized Root Mean Square Residual; Invariance assessed by Robust (Satorra-Bentler) chi-square different test (Δχ^2^) calculated from log likelihood, absolute change in the CFI (ΔCFI), RMSEA (ΔRMSEA), and SRMR (ΔSRMR) between invariance models.*

#### Invariance Across Age Groups

The middle section of Table 6 presents the measurement invariance tests of the SOAR Scale across age (18–34 vs. 35–54, vs. 55–74). Results found that only the configural model fit the data well according to the study model fit criteria. These results imply that only the four-factor structure and loading-patterns of items of the SOAR Scale is the same across age groups, and it may not be possible to meaningfully investigate mean differences and comparability in the factors across age groups (Putnick and Bornstein, 2016; Wang et al., 2018).

#### Invariance Across Education Groups

The bottom section of Table 6 presents the measurement invariance tests of the SOAR Scale scores across education (undergraduates vs. graduates). Results found metric, scalar, strict, latent variance-covariance, and latent means invariance. Latent means on the first-order factors of the SOAR Scale were compared across education in the strict invariance model. Results found latent means between education levels in the Opportunities, Aspirations, and Results factors were *p* > 0.05, and finding latent means for these factors did not meaningfully differ across education. However, latent means in the Strengths factor for graduates (Δ x̄ = 0.31, *SE* = 0.15, *p* = 0.04) were statistically different from undergraduates. Taken together, these findings imply that valid comparisons in latent means across education may be possible for the Opportunities, Aspirations, and Results factors of the SOAR Scale.

## Discussion

This study aimed to investigate the psychometric properties and measurement invariance of the SOAR Scale in a sample of 295 U.S. working professionals across gender, age, and education groups using CFA and ESEM measurement models. SOAR is a strengths-based framework for strategic thinking, planning, conversations, and leading that focuses on strengths, opportunities, aspirations, and results. The 12-item SOAR Scale was designed to measure four first-order factors representing the four elements of SOAR: Strengths (Strengths, Assets, Capabilities), Opportunities (Opportunities, Ideas, Possibilities), Aspirations (Aspirations, Ideas, Possibilities), and Results (Results, Completed Tasks, Outcomes). We selected items to support *a priori* assumptions about the SOAR framework theory and hypothesized the pattern of item loadings onto their respective factors. EFA found the 12 items loaded on their *a priori* factors. Competing structural equation measurement models found that the ESEM model comprised of the Strengths, Opportunities, Aspirations, and Results latent factors had the best model fit based on goodness-of-fit indexes and measurement quality (e.g., significant loadings with λ > 0.35 and expected pattern of loadings), item uniqueness (e.g., δ > 0.10 but < 0.90), cross-loadings (e.g., λ < 0.5), and factor correlations (e.g., ESEM had smaller factorial intercorrelations between latent factors than ICM CFA). Measurement invariance tests found scalar invariance of the SOAR Scale across gender and education groups.

### Psychometric Properties of the Strengths, Opportunities, Aspirations, and Results Scale

Results support the reliability of the SOAR Scale in terms of Cronbach’s alpha and composite reliability. Results of ESEM as the best fitting solution (model 4) support the construct validity of the SOAR Scale in terms of model fit criteria and measurement quality (e.g., significant loadings with λ > 0.35 and expected pattern of loadings), item uniqueness (e.g., δ > 0.10 but < 0.90), and cross-loadings (e.g., λ < 0.2). The Hierarchical CFA and ESEM solutions did not have better model fit than their respective first-order models (i.e., models 1 and 4, respectively), suggesting the absence of a hierarchical structure of the SOAR Scale in which the four first-order factors load onto a second-order SOAR factor. Although the Bifactor ESEM had a slightly better model fit than the ESEM, an examination of the measurement quality of the Bifactor ESEM rejected its consideration as an acceptable representation of the data solution due to the absence of a well-defined general and specific factors.

These results support the construct validity of the SOAR Scale as a 12-item scale that validly measures four first-order factors: Strengths, Opportunities, Aspirations, and Results. SOAR is conceptualized as a strengths-based framework for strategic thinking, planning, conversations, and leading (Cole and Stavros, 2019), in which strengths refer to an individual’s “natural capacity for behaving, thinking, or feeling in a way that allows optimal functioning and performance in the pursuit of value outcomes” (Linley and Harrington, 2006, p. 88). In managing personal strengths, “Strengths [sic] do more than perform, they transform—strengths are what make us feel stronger and therefore magnify ‘what is best’ and imagine ‘what is next’ in order to create upward spirals. We live in a universe of strengths—the wider the lens, the better the view” (Cooperrider and Godwin, 2011, p. 744).

Gallup Poll data related to strengths development suggests the most effective people know their strengths and aspire to leverage strengths for success (Rath, 2007). In management, the strengths revolution has taught leaders to focus on what they and their teams do best, rather than weaknesses (Ludema and Johnson, 2018). Once individuals are clear on their strengths, the next step is about being intentional and aspirational about personal or professional desires. Knowing one’s aspirations is about being clear about intentions for success. When aspirations are clear, strengths can be leveraged and aligned with other resources to achieve optimal success (Cameron, 2013).

### Measurement Invariance of the Strengths, Opportunities, Aspirations, and Results Scale

Measurement invariance of a measurement tool is a logical requirement for the evaluation of substantive hypotheses, such as mean difference between groups or effects across groups (Vandenberg and Lance, 2000; Millsap, 2012). We examined the measurement invariance of the SOAR Scale across gender, age, and education groups in the ESEM best fitting model. Whereas the SOAR Scale was non-invariant across age, scalar invariance was established across gender and was fully invariant across education groups. Thus, the SOAR Scale may be used for investigating meaningful differences in SOAR factors across participants with differing levels of education, but additional research is required to examine the measurement invariance of the SOAR Scale across gender and age.

### Implications for Researchers and Practitioners

Tests of the psychometric properties of the SOAR Scale provide strong statistical support for its construct validity. Researchers and practitioners should use the SOAR Scale to obtain scores in the factors of Strengths, Opportunities, Aspirations, and Results for the rapid assessment of natural capacity for strategic thinking, planning, conversations, and leading from a SOAR-based, generative perspective. The empirical research by positive organizational scholars confirms that positive states of being, organizing, and leading improve organizational performance, individual physiological health, and wellbeing (Cameron, 2013, 2021). Researchers can use the SOAR Scale to investigate the SOAR factors and their relationship to a variety of individual, team, and organizational outcomes, such as leadership style, leadership effectiveness, employee engagement, collaboration, wellbeing, and resilience. For example, empirical research could investigate the SOAR factors as predictors of resilience by having participants complete the SOAR Scale along with a battery of self-report measures on resilience, such as the Satisfaction With Life Scale (Biswas-Diener et al., 2011), the Subjective Happiness Scale (Lyubomirsky and Lepper, 1999), the Adult Hope Scale (Snyder et al., 1991), the Grit Scale (Duckworth et al., 2007), the Connor-Davidson Resilience Scale (Campbell-Sills and Stein, 2007), the Burnout Assessment Tool (Schaufeli et al., 2020), and the Utrecht Work Engagement Survey (Schaufeli et al., 2006). Researchers can also use the SOAR Scale to investigate various antecedents to the SOAR factors, such as self-reported emotional intelligence (Wong and Law, 2002; Jordan and Lawrence, 2009), leadership style (Podsakoff et al., 1990), team cohesion and communication, and various organizational characteristics (e.g., workplace culture). Finally, researchers can use the SOAR Scale to investigate meaningful differences in the SOAR factor scores across various groups, for example, in studies of the differences in SOAR-based capacity between leaders and followers.

Organizations need strategic thinkers that can make sure goals and objectives align with strategies, missions, and visions to ensure success. Strategic planning needs to map out the direction, purpose, position, strategies, tactics, and resources to achieve the goals and objectives. Strategic individuals need to think and plan intentionally by understanding how the complex relationship between the organization’s capabilities and its environment influence decisions that may have a positive impact on organizational success. They must facilitate conversations with a variety of stakeholders, and this includes asking the right questions (Center for Creative Leadership, 2020). For practitioners, the SOAR Scale can be used in training and coaching sessions to help individuals understand their natural capacity to think and plan from a SOAR-based perspective by examining their SOAR Scale factor scores. This will help with individual and team development, performance, communication, and shared visioning of a positive future (Driver, 2011; Hawkins, 2014).

### Limitations and Future Directions

The present study makes a significant contribution to the area of positive psychological assessment measures by presenting the psychometric properties of the SOAR Scale, a brief 12-item rapid assessment measure of Strengths, Opportunities, Aspirations, and Results. This study does have some specific limitations. First, although full invariance of the SOAR Scale was found across education, the relatively small sample size of this study for structural equation modeling research may have limited the ability of the measurement invariance tests to find invariance of the SOAR Scale across gender and age. This is of particular importance for research on investigating the existence of substantive differences in SOAR for men and women and across generational differences (e.g., different age groups). Future research should reexamine measurement invariance of the SOAR Scale across gender and age groups. We also suggest that future research should examine the use of the SOAR Scale as a measure of SOAR across other groups, such as leaders vs. followers (with leader-follower matching if possible). Future research should also have followers complete a revised SOAR Scale with items modified to reflect focus on SOAR by the leader (Libbrecht et al., 2010; Elfenbein et al., 2015; Koh and O’Higgins, 2018). For example, items could be modified as “When your leader approaches strategy, how often do they focus on strengths?” This could also be used in teams.

Finally, the study is limited by the absence of testing for other forms of validity beyond construct, namely convergent, discriminant, and predictive validity. While there are no other tools that directly measure SOAR, future research should examine the convergent validity of the SOAR Scale with other strengths-based measures, such as the Strengths-Use Scale (Govindji and Linley, 2007). Future research should examine the discriminant validity of the SOAR Scale in participants who also complete self-report measures of theoretically unrelated constructs. Finally, future research should examine the predictive validity of the SOAR Scale in participants who also complete self-report measures of outcomes likely to be impacted by SOAR, such as self-reported resilience or wellbeing.

## Conclusion

Given the current business and economic climate in which there are technological changes, environmental changes due to the pandemic and the climate, rapid changing of skillsets in the workplace, employment shortages and turnover, and generational differences in the workforce, we need to have strategic thinkers, planners, and leaders who can surface strengths, create shared aspirations, identify opportunities, and build capabilities with a results-driven focus (Isern and Pung, 2007; Bachmann et al., 2021). The SOAR Scale, a rapid 12-item self-report survey with demonstrated reliability and construct validity, can help individuals, researchers, and practitioners leverage survey scores in the Strengths, Aspirations, Opportunities, and Results factors to create a strategy for optimal individual, team, or organization performance based on the awareness of strengths and aspirations and the identification of opportunities for growth with meaningful and measurable results.

## Data Availability Statement

The original contributions presented in the study are included in the article/Supplementary Material, further inquiries can be directed to the corresponding author/s.

## Ethics Statement

The studies involving human participants were reviewed and approved by Lawrence Technological University Institutional Review Board. The patients/participants provided their written informed consent to participate in this study.

## Author Contributions

All the authors made substantial contributions to the whole study. MC and JS developed the idea and designed the study. JC helped with the data collection and the analysis. AS helped with the data analysis.

## Conflict of Interest

The authors declare that the research was conducted in the absence of any commercial or financial relationships that could be construed as a potential conflict of interest.

## Publisher’s Note

All claims expressed in this article are solely those of the authors and do not necessarily represent those of their affiliated organizations, or those of the publisher, the editors and the reviewers. Any product that may be evaluated in this article, or claim that may be made by its manufacturer, is not guaranteed or endorsed by the publisher.

## Footnotes

1
http://appreciativeinquiry.case.edu/intro/vision.cfm

2
www.etymonline.com

## References

[B1] Ames M. Runco M. A. (2005). Predicting entrepreneurship from ideation and divergent thinking. *Creat. Innov. Manag.* 14 311–315. 10.1111/j.1467-8691.2004.00349.x

[B2] Asparouhov T. Muthén B. (2009). Exploratory structural equation modeling. *Struct. Equ. Modeling* 16 397–438. 10.1080/10705510903008204

[B3] Bachmann H. Skerritt D. Mcnally E. Y. (2021). *How Capability Building Can Power Transformation.* New York, NY: McKinsey & Company.

[B4] Baum J. R. Locke E. A. Kirkpatrick S. A. (1998). A longitudinal study of the relation of vision and vision communication to venture growth in entrepreneurial firms. *J. Appl. Psychol.* 83 43–54. 10.1037/0021-9010.83.1.43

[B5] Beatty K. C. Hughes R. L. (2005). Strategic aims: making the right moves in leadership. *Leadersh. Act.* 25 3–6. 10.1002/lia.1123

[B6] Bhatnagar V. R. (2020). Conceptualizing employee strengths at work and scale development. *Manag. Res. Rev.* 43 1273–1288. 10.1108/MRR-08-2019-0367

[B7] Binnendijk A. (2001). *Results Based Management In The Development Cooperation Agencies: A Review Of Experience.* Paris: DAC Working Party on Aid Evaluation.

[B8] Biswas-Diener R. Kashdan T. B. Minhas G. (2011). A dynamic approach to psychological strength development and intervention. *J. Positive Psychol.* 6 106–118. 10.1080/17439760.2010.545429

[B9] Boccagni P. (2017). Aspirations and the subjective future of migration: comparing views and desires of the “time ahead” through the narratives of immigrant domestic workers. *Comp. Migr. Stud.* 5:4. 10.1186/s40878-016-0047-6 28251058 PMC5306300

[B10] Bolman L. Deal T. (2008). *Reframing Organizations: Artistry, Choice, And Leadership.* San Francisco, CA: Jossey-Bass.

[B11] Brownlee K. Rawana J. Franks J. Harper J. Bajwa J. O’brien E. (2013). A systematic review of strengths and resilience outcome literature relevant to children and adolescents. *Child Adolesc. Soc. Work J.* 30 435–459. 10.1007/s10560-013-0301-9

[B12] Brunetto Y. Dick T. Xerri M. Cully A. (2019). Building capacity in the healthcare sector: a strengths-based approach for increasing employees’ well-being and organisational resilience. *J. Manag. Org.* 26 309–323. 10.1017/jmo.2019.53

[B13] Bryson J. M. Crosby B. C. Bryson J. K. (2009). Understanding strategic planning and the formulation and implementation of strategic plans as a way of knowing: the contributions of actor-network theory. *Int. Public Manag. J.* 12 172–207. 10.1080/10967490902873473

[B14] Burke R. J. (2002). Do workaholics prefer demanding, aggressive, and results-oriented organizational cultures? *Career Dev. Int.* 7 211–217. 10.1108/13620430210431299

[B15] Burkus D. (2011). Building the strong organization: exploring the role of organizational design in strengths-based leadership. *J. Strateg. Leadersh.* 3 54–66.

[B16] Bushe G. R. (2007). Appreciative inquiry is not (just) about the positive. *OD Pract.* 39 33–38.

[B17] Bushe G. R. (2013). “Generative process, generative outcome: the transformational potential of appreciative inquiry,” in *Organizational Generativity: The Appreciative Inquiry Summit And A Scholarship Of Transformation*, eds Cooperrider D. L. Zandee D. P. Godwin L. N. Avital M. Boland B. (Bingley: Emerald Group Publishing Limited), 89–113. 10.1016/j.cpr.2011.06.003

[B18] Bushe G. R. Marshak R. J. (2014). “Dialogic organization development,” in *The NTL Handbook Of Organization Development And Change*, 2nd Edn, eds Jones B. B. Brazzel M. (San Francisco, CA: Wiley), 193–211. 10.1002/9781118836170.ch10

[B19] Cameron K. (2021). *Positive Energizing Leadership: Virtuous Actions And Relationships That Create High Performance.* Oakland, CA: Berrett-Koehler Publishers.

[B20] Cameron K. S. (2013). *Practicing Positive Leadership: Tools And Techniques That Create Extraordinary Results.* San Francisco, CA: Berrett-Koehler Publishers.

[B21] Cameron K. S. Spreitzer G. M. (2012). *The Oxford Handbook Of Positive Organizational Scholarship.* New York, NY: Oxford University Press.

[B22] Cameron K. S. Dutton J. E. Quinn R. E. (2003). *Positive Organizational Scholarship: Foundations Of A New Discipline.* San Francisco, CA: Berrett-Koehler.

[B23] Cameron K. Lavine M. (2006). *Making The Impossible Possible: Leading Extraordinary Performance: The Rocky Flats Story.* San Francisco, CA: Berrett-Koehler Publishers.

[B24] Campbell-Sills L. Stein M. B. (2007). Psychometric analysis and refinement of the connor–davidson resilience scale (cd-risc): validation of a 10-item measure of resilience. *J. Traumatic Stress* 20 1019–1028. 10.1002/jts.20271 18157881

[B25] Center for Creative Leadership (2020). *How To Become A Strategic Leader.* Greensboro, NC: Center for Creative Leadership Inc.

[B26] Chaffee E. E. (1985). Three models of strategy. *Acad. Manag. Rev.* 10 89–98. 10.5465/amr.1985.4277354

[B27] Chen F. F. (2007). Sensitivity of goodness of fit indexes to lack of measurement invariance. *Struct. Equ. Modeling* 14 464–504. 10.1080/10705510701301834

[B28] Christensen C. M. (1997). Marketing strategy: learning by doing. *Harvard Bus. Rev.* 75 141–151. 10174795

[B29] Clifton D. O. Harter J. K. (2003). “Investing in strengths,” in *Positive Organizational Scholarship: Foundations Of A New Discipline*, eds Cameron K. S. Dutton J. E. Quinn R. E. (Oakland, CA: Berrett-Kohler), 111–121.

[B30] Colbert B. A. Kurucz E. C. (2007). Three conceptions of triple bottom line business sustainability and the role for hrm. *HR Hum. Resour. Plan.* 30 21–29.

[B31] Cole M. L. Stavros J. M. (2019). “Soar: a framework to build positive psychological capacity in strategic thinking, planning, and leading,” in *Theoretical Approaches To Multi-Cultural Positive Psychological Interventions*, eds Van Zyl L. E. Rothmann S. Sr. (Cham: Springer Nature), 505–521. 10.1007/978-3-030-20583-6_23

[B32] Collins J. C. Porras J. I. (1996). Building your company’s vision. *Harvard Bus. Rev.* 74 65–77.

[B33] Collins J. M. (2001). *Good To Great: Why Some Companies Make The Leap And Others Don’t.* New York, NY: HarperCollins Publishers Inc.

[B34] Cooperrider D. L. Godwin L. (2011). “Positive organization development: Innovation-inspired change in an economy and ecology of strengths,” in *The Oxford Handbook Of Positive Organizational Scholarship*, eds Cameron K. Spreitzer G. (Oxford: Oxford University Press), 737–750.

[B35] Cooperrider D. Whitney D. (2000). “A positive revolution in change: appreciative inquiry,” in *Appreciative Inquiry*, eds Cooperrider D. Sorensen P. F. Whitney D. Yeager T. F. (Champaign, IL: Stipes), 3–28.

[B36] Cravens K. S. Oliver E. G. Stewart J. S. (2010). Can a positive approach to performance evaluation help accomplish your goals? *Bus. Horizons* 53 269–279. 10.1016/j.bushor.2009.09.005

[B37] Danışman I. G. Tiftik N. (2014). Measuring family strengths and capabilities: reliability and validity of the turkish version of the family functioning style scale. *Proc. Soc. Behav. Sci.* 114 346–350. 10.1016/j.sbspro.2013.12.709

[B38] Daulon S. Greiner M. Hudson J. Jolliff T. Kerr J. Thatchenkery T. (2017). “Application of appreciative inquiry at a municipal social services agency: a case study,” in *Knowledge Creation And Organizational Well-Being: Leveraging Talent And Appreciative Intelligence*, eds Sardana G. D. Thatchenkery T. (New Delhi: Bloomsbury), 3–14.

[B39] Davidsson P. Grégoire D. A. Lex M. (2021). Venture idea assessment (via): development of a needed concept, measure, and research agenda. *J. Bus. Ventur.* 36:106130. 10.1016/j.jbusvent.2021.106130

[B40] De Beer L. T. Morin A. J. S. (2022). (*b*)*Esem Invariance Syntax Generator For Mplus.* Available online at: https://statstools.app/b_esem/ (accessed February 24, 2022).

[B41] De Beer L. T. Van Zyl L. E. (2019). *Esem Code Generator For Mplus.* Available online at: https://www.surveyhost.co.za/esem/ (accessed February 24, 2022).

[B42] Deci E. L. Ryan R. M. (1985). *Intrinsic Motivation And Self-Determination In Human Behavior.* New York, NY: Plenum.

[B43] Deci E. L. Ryan R. M. (2000). The “what” and “why” of goal pursuits: human needs and the self-determination of behavior. *Psychol. Inq.* 11 227–268. 10.1207/S15327965PLI1104_01

[B44] Ding H. Yu E. Li Y. (2020). Strengths-based leadership and its impact on task performance: a preliminary study. *South African J. Bus. Manag.* 51 1–9. 10.4102/sajbm.v51i1.1832

[B45] Driver M. (2011). *Coaching Positively: Lessons For Coaches From Positive Psychology.* Berkshire: Open University Press.

[B46] Dubreuil P. Ben Mansour J. Forest J. Courcy F. Fernet C. (2021). Strengths use at work: positive and negative emotions as key processes explaining work performance. *Can. J. Adm. Sci.* 38 150–161. 10.1002/cjas.1595

[B47] Duckworth A. L. Peterson C. Matthews M. D. Kelly D. R. (2007). Grit: perseverance and passion for long-term goals. *J. Pers. Soc. Psychol.* 92 1087–1101. 10.1037/0022-3514.92.6.1087 17547490

[B48] Dutton J. E. Fahey L. Narayanan V. K. (1983). Toward understanding strategic issue diagnosis. *Strateg. Manag. J.* 4 307–323. 10.1002/smj.4250040403

[B49] Eckhardt J. T. Shane S. A. (2003). Opportunities and entrepreneurship. *J. Manag.* 29 333–349. 10.1177/014920630302900304

[B50] Edmondson A. C. (2019). *The Fearless Organization: Creating Psychological Safety In The Workplace For Learning, Innovation, And Growth.* Hoboken, NJ: John Wiley & Sons, Inc.

[B51] Elfenbein H. A. Barsade S. G. Eisenkraft N. (2015). The social perception of emotional abilities: expanding what we know about observer ratings of emotional intelligence. *Emotion* 15 17–34. 10.1037/a0038436 25664949

[B52] Elkington J. (1998). Accounting for the triple bottom line. *Meas. Bus. Excellence* 2 18–22. 10.1108/eb025539

[B53] Fisher S. Zapolski T. B. Wheeler L. Arora P. G. Barnes-Najor J. (2020). Multigroup ethnic identity measurement invariance across adolescence and diverse ethnic groups. *J. Adolesc.* 83 42–51. 10.1016/j.adolescence.2020.07.006 32711160 PMC7906292

[B54] Fredrickson B. L. (1998). What good are positive emotions? *Rev. Gen. Psychol.* 2 300–319. 10.1037/1089-2680.2.3.300 21850154 PMC3156001

[B55] Fredrickson B. L. (2003a). “Positive emotions and upward spirals in organizations,” in *Positive Organizational Scholarship*, eds Cameron K. S. Dutton J. E. Quinn R. E. (San Francisco, CA: Berrett-Kohler), 163–175.

[B56] Fredrickson B. L. (2003b). The value of positive emotions. *Am. Sci.* 91 330–335. 10.1511/2003.4.330

[B57] Fredrickson B. L. (2004). The broaden-and-build theory of positive emotions. *Philos. Trans. R. Soc. Lond. Ser. B* 359 1367–1377. 10.1098/rstb.2004.1512 15347528 PMC1693418

[B58] Fredrickson B. L. (2009a). *Positivity: Groundbreaking Research Reveals How To Embrace The Hidden Strength Of Positive Emotions, Overcome Negativity, And Thrive.* New York, NY: Crown Publishers/Random House.

[B59] Fredrickson B. L. (2009b). *Positivity: Top-Notch Research Reveals The Upward Spiral That Will Change Your Life.* New York, NY: Three Rivers Press.

[B60] Fredrickson J. W. (1985). Effects of decision motive and organizational performance level on strategic decision processes. *Acad. Manag. J.* 28 821–843. 10.5465/256239

[B61] Frieden G. (2019). “Chapter 17 – screening for strengths and assets in adolescents,” in *Adolescent Health Screening: An Update In The Age Of Big Data*, ed. Morelli V. (St. Louis, MS: Elsevier), 227–243. 10.1016/B978-0-323-66130-0.00017-X

[B62] Ghielen S. T. S. Van Woerkom M. Christina Meyers M. (2018). Promoting positive outcomes through strengths interventions: a literature review. *J. Positive Psychol.* 13 573–585. 10.1080/17439760.2017.1365164

[B63] Govindji R. Linley P. A. (2007). Strengths use, self-concordance and well-being: Implications for strengths coaching and coaching psychologists. *Int. Coach. Psychol. Rev.* 2 143–153. 10.1037/t54221-000

[B64] Haller A. (1968). On the concept of aspiration. *Rural Sociol.* 33 484–487.

[B65] Hamel G. Prahalad C. K. (2005). Strategic intent. *Harvard Bus. Rev.* 83 148–161. 10303477

[B66] Hamel G. Doz Y. L. Prahalad C. K. (1989). Collaborate with your competitors and win. *Harvard Bus. Rev.* 67 133–139.

[B67] Hart C. S. (2016). How do aspirations matter? *J. Hum. Dev. Capabil.* 17 324–341. 10.1080/19452829.2016.1199540

[B68] Hart S. L. (1992). An integrative framework for strategy-making processes. *Acad. Manag. Rev.* 17 327–351. 10.5465/amr.1992.4279547

[B69] Harzer C. Ruch W. (2013). The application of signature character strengths and positive experiences at work. *J. Happiness Stud.* 14 965–983. 10.1007/s10902-012-9364-0

[B70] Hawkins P. (2014). *Leadership Team Coaching: Developing Collective Transformational Leadership*, 2nd Edn. London: Kogan Page Limited.

[B71] Helfat C. E. Peteraf M. A. (2003). The dynamic resource-based view: capability lifecycles. *Strategic Manag. J.* 24 997–1010. 10.1002/smj.332

[B72] Helms M. M. Nixon J. (2010). Exploring swot analysis – where are we now? *J. Strategy Manag.* 3 215–251. 10.1108/17554251011064837

[B73] Hinrichs G. (2010). Soaring for sustainability. *AI Pract.* 12 31–36.

[B74] Holma T. Lehtimäki H. Thatchenkery T. (2017). Positive approaches to enhance customer-focused knowledge sharing culture in a financial services organisation. *Int. J. Hum. Resour. Dev. Manag.* 17 21–36. 10.1504/ijhrdm.2017.085273 35009967

[B75] Huang H.-C. (2009). Designing a knowledge-based system for strategic planning: a balanced scorecard perspective. *Expert Syst. Appl.* 36 209–218. 10.1016/j.eswa.2007.09.046

[B76] Hubbard G. (2009). Measuring organizational performance: beyond the triple bottom line. *Bus. Strategy Environ.* 18 177–191. 10.1002/bse.564

[B77] Hurley A. E. Scandura T. A. Schriesheim C. A. Brannick M. T. Seers A. Vandenberg R. J. (1997). Exploratory and confirmatory factor analysis: guidelines, issues, and alternatives. *J. Org. Behav.* 18 667–683. 10.1002/(sici)1099-1379(199711)18:6<667::aid-job874>3.0.co;2-t

[B78] Isern J. Pung C. (2007). Driving radical change. *McKinsey Q.* 4 24–35.

[B79] Jackson S. E. Dutton J. E. (1988). Discerning threats and opportunities. *Adm. Sci. Q.* 33 370–387. 10.2307/2392714

[B80] Jamali D. (2006). Insights into triple bottom line integration from a learning organization perspective. *Bus. Process Manag. J.* 12 809–821. 10.1108/14637150610710945

[B81] Jordan P. J. Lawrence S. A. (2009). Emotional intelligence in teams: Development and initial validation of the short version of the workgroup emotional intelligence profile (weip-s). *J. Manag. Org.* 15 452–465. 10.5172/jmo.15.4.452

[B82] Kaiser H. F. (1974). An index of factorial simplicity. *Psychometrika* 39 31–36. 10.1207/s15327906mbr1901_1 26776065

[B83] Kaplan R. S. Norton D. P. (2006). *The Balanced Scorecard: Translating Strategy Into Action.* Boston, MA: Harvard Business School Press.

[B84] Kasser T. Ryan R. M. (1993). A dark side of the american dream: correlates of financial success as a central life aspiration. *J. Pers. Soc. Psychol.* 65 410–422. 10.1037/0022-3514.65.2.410 8366427

[B85] Kasser T. Ryan R. M. (2001). “Be careful what you wish for: optimal functioning and the relative attainment of intrinsic and extrinsic goals,” in *Life Goals And Well-Being: Towards A Positive Psychology Of Human Striving*, eds Schmuck P. Sheldon K. M. (Ashland, OH: Hogrefe & Huber Publishers), 116–131.

[B86] Kienfie Liau A. Chow D. Teck Kiang T. Senf K. (2010). Development and validation of the personal strengths inventory using exploratory and confirmatory factor analyses. *J. Psychoeduc. Assess.* 29 14–26. 10.1177/0734282910365648

[B87] Kim H.-Y. (2013). Statistical notes for clinical researchers: assessing normal distribution (2) using skewness and kurtosis. *Restor. Dent. Endod.* 38 52–54. 10.5395/rde.2013.38.1.52 23495371 PMC3591587

[B88] Kline R. B. (2015). *Principles And Practices Of Structural Equation Modelling*, 4th Edn. New York, NY: Guilford Press.

[B89] Koh C. B. O’Higgins E. (2018). Relationships between emotional intelligence, perceived and actual leadership effectiveness in the military context. *Military Psychol.* 30 27–42. 10.1080/08995605.2017.1419021

[B90] Laszlo C. Cooperrider D. L. (2010). “Creating sustainable value: a strength-based whole system approach,” in *Positive Design And Appreciative Construction: From Sustainable Development To Sustainable Value*, Vol. 3 eds Thatchenkery T. Cooperrider D. L. Avital M. (Bingley: Emerald Group Publishing Limited), 17–33. 10.1108/S1475-915220100000003006

[B91] Lavy S. Littman-Ovadia H. (2016). My better self: using strengths at work and work productivity, organizational citizenship behavior, and satisfaction. *J. Career Dev.* 44 95–109. 10.1177/0894845316634056

[B92] Li C.-H. (2016). Confirmatory factor analysis with ordinal data: comparing robust maximum likelihood and diagonally weighted least squares. *Behav. Res. Methods* 48 936–949. 10.3758/s13428-015-0619-7 26174714

[B93] Libbrecht N. Lievens F. Schollaert E. (2010). Measurement equivalence of the wong and law emotional intelligence scale across self and other ratings. *Educ. Psychol. Meas.* 70 1007–1020. 10.1177/0013164410378090

[B94] Liedtka J. M. (1998). Strategic thinking: can it be taught? *Long Range Plan.* 31 120–129. 10.1016/s0024-6301(97)00098-8

[B95] Linley P. A. Harrington S. (2006). Playing to your strengths. *Psychologist* 19 86–89.

[B96] Littman-Ovadia H. Steger M. (2010). Character strengths and well-being among volunteers and employees: toward an integrative model. *J. Positive Psychol.* 5 419–430. 10.1080/17439760.2010.516765

[B97] Locke E. A. Latham G. P. (1990). *A Theory Of Goal Setting & Task Performance.* Englewood Cliffs, NJ: Prentice Hall.

[B98] Locke E. A. Cartledge N. Koeppel J. (1968). Motivational effects of knowledge of results: a goal-setting phenomenon? *Psychol. Bull.* 70 474–485. 10.1037/h0026737

[B99] Ludema J. Johnson A. (2018). *Marcus Buckingham, Founder Of ‘The Strengths Revolution,’ On The Value Of Excellence. Forbes [Online].* Available online at: https://www.forbes.com/sites/amberjohnson-jimludema/2018/05/09/marcus-buckingham-and-the-value-of-excellence/ (accessed January 13, 2022).

[B100] Lyubomirsky S. Lepper H. S. (1999). A measure of subjective happiness: preliminary reliability and construct validation. *Soc. Indic. Res.* 46 137–155. 10.1023/A:1006824100041

[B101] Miglianico M. Dubreuil P. Miquelon P. Bakker A. B. Martin-Krumm C. (2020). Strength use in the workplace: a literature review. *J. Happiness Stud.* 21 737–764. 10.1007/s10902-019-00095-w

[B102] Millsap R. E. (2012). *Statistical Approaches To Measurement Invariance.* New York, NY: Routledge.

[B103] Mintzberg H. (1973). *The Nature Of Managerial Work.* New York, NY: Harper & Row.

[B104] Moore S. L. Ellsworth J. B. Kaufman R. (2011). Visions and missions: are they useful? A quick assessment. *Perform. Improv.* 50 15–24. 10.1002/pfi.20222

[B105] Morin A. J. S. Arens A. K. Marsh H. W. (2016). A bifactor exploratory structural equation modeling framework for the identification of distinct sources of construct-relevant psychometric multidimensionality. *Struct. Equ. Modeling* 23 116–139. 10.1080/10705511.2014.961800

[B106] Morin A. J. S. Myers N. D. Lee S. (2020). “Modern factor analytic techniques: Bifactor models, exploratory structural equation modeling (esem), and bifactor−esem,” in *Handbook Of Sport Psychology*, 4th Edn, eds Tenenbaum G. Eklund R. C. (Hoboken, NJ: John Wiley & Sons, Inc), 1044–1073. 10.1002/9781119568124.ch51

[B107] Muthén B. O. Asparouhov T. (2016). “Multi-dimensional, multi-level, and multi-timepoint item response modeling,” in *Handbook Of Item Response Theory*, ed. Van Der Linden W. J. (Boca Raton, FL: CRC Press), 527–539.

[B108] Muthén L. K. Muthén B. O. (2017). *Mplus User’s Guide Version 8.* Los Angeles, CA: Muthen & Muthen.

[B109] Nunnally J. C. Bernstein I. H. (1994). *Psychometric Theory.* New York, NY: McGraw-Hill.

[B110] Park N. Peterson C. (2006). Moral competence and character strengths among adolescents: the development and validation of the values in action inventory of strengths for youth. *J. Adolesc.* 29 891–909. 10.1016/j.adolescence.2006.04.011 16766025

[B111] Park N. Peterson C. Seligman M. E. P. (2004). Strengths of character and well-being. *J. Soc. Clin. Psychol.* 23 603–619. 10.1521/jscp.23.5.603.50748

[B112] Peláez M. J. Coo C. Salanova M. (2020). Facilitating work engagement and performance through strengths-based micro-coaching: a controlled trial study. *J. Happiness Stud.* 21 1265–1284. 10.1007/s10902-019-00127-5

[B113] Perugini M. Bagozzi R. P. (2004). The distinction between desires and intentions. *Eur. J. Soc. Psychol.* 34 69–84. 10.1002/ejsp.186

[B114] Podsakoff P. M. Mackenzie S. Moorman R. Fetter R. (1990). Transformational leader behaviors and their effects on followers’ trust in leader, satisfaction, and organizational citizenship behaviors. *Leadersh. Q.* 1 107–142. 10.1016/1048-9843(90)90009-7

[B115] Porter M. E. (1980). *Competitive Strategy.* New York, NY: Free Press.

[B116] Prahalad C. K. Hamel G. (1994). Strategy as a field of study: why search for a new paradigm? *Strateg. Manag. J.* 15 5–16. 10.1002/smj.4250151002 21882411

[B117] Putnick D. L. Bornstein M. H. (2016). Measurement invariance conventions and reporting: the state of the art and future directions for psychological research. *Dev. Rev.* 41 71–90. 10.1016/j.dr.2016.06.004 27942093 PMC5145197

[B118] Puyt R. Lie F. B. De Graaf F. J. Wilderom C. P. M. (2020). Origins of swot analysis. *Acad. Manag. Proc.* 2020:17416. 10.5465/AMBPP.2020.132

[B119] Rath T. (2007). *Strengthsfinder 2.0.* New York, NY: Simon and Schuster.

[B120] Reise S. P. (2012). The rediscovery of bifactor measurement models. *Multivariate Behav. Res.* 47 667–696. 10.1080/00273171.2012.715555 24049214 PMC3773879

[B121] Ressler C. Thompson J. (2008). *Why Work Sucks And How To Fix It.* New York, NY: Penguin Group.

[B122] Rudnev M. Lytkina E. Davidov E. Schmidt P. Zick A. (2018). Testing measurement invariance for a second-order factor. A cross-national test of the alienation scale. *Methods Data Anal.* 12 47–76. 10.12758/mda.2017.11

[B123] Salmoni A. W. Schmidt R. A. Walter C. B. (1984). Knowledge of results and motor learning: a review and critical reappraisal. *Psychol. Bull.* 95 355–386. 10.1037/0033-2909.95.3.355 6399752

[B124] Santalainen T. J. Hunt J. G. (1988). Change differences from an action research, results-oriented od program in high and low-performing finnish banks. *Group Org. Stud.* 13 413–440. 10.1177/105960118801300402

[B125] Satorra A. Bentler P. M. (2010). Ensuring positiveness of the scaled difference chi-square test statistic. *Psychometrika* 75 243–248. 10.1007/s11336-009-9135-y 20640194 PMC2905175

[B126] Scales P. C. (2011). Youth developmental assets in global perspective: results from international adaptations of the developmental assets profile. *Child Indic. Res.* 4 619–645. 10.1007/s12187-011-9112-8

[B127] Schaufeli W. B. Bakker A. B. Salanova M. (2006). The measurement of work engagement with a short questionnaire: a cross-national study. *Educ. Psychol. Meas.* 66 701–716. 10.1177/0013164405282471

[B128] Schaufeli W. B. Desart S. De Witte H. (2020). Burnout assessment tool (bat)—development, validity, and reliability. *Int. J. Environ. Res. Public Health* 17 1–21. 10.3390/ijerph17249495 33352940 PMC7766078

[B129] Scheaf D. J. Loignon A. C. Webb J. W. Heggestad E. D. Wood M. S. (2020). Measuring opportunity evaluation: conceptual synthesis and scale development. *J. Bus. Ventur.* 35:105935. 10.1016/j.jbusvent.2019.04.003

[B130] Seiling J. Hinrichs G. (2005). Mindfulness and constructive accountability as critical elements of effective sensemaking: a new imperative for leaders as sensemanagers. *Org. Dev. J.* 23 82–88.

[B131] Sekerka L. E. Comer D. R. Godwin L. N. (2014). Positive organizational ethics: cultivating and sustaining moral performance. *J. Bus. Ethics* 119 435–444. 10.1007/s10551-013-1911-z

[B132] Seligman M. E. P. (2002). “Positive psychology, positive prevention, and positive therapy,” in *Handbook Of Positive Psychology*, eds Snyder C. R. Lopez S. J. (New York, NY: Oxford University Press), 3–9.

[B133] Seligman M. E. P. Csikszentmihalyi M. (2000). Positive psychology: an introduction. *Am. Psychol.* 55 5–14. 10.1037/0003-066X.55.1.5 11392865

[B134] Seligman M. E. P. Steen T. A. Park N. Peterson C. (2005). Positive psychology progress: empirical validation of interventions. *Am. Psychol.* 60 410–421. 10.1037/0003-066X.60.5.410 16045394

[B135] Senge P. (1990). *The Fifth Discipline.* New York, NY: Doubleday.

[B136] Shane S. Venkataraman S. (2000). The promise of entrepreneurship as a field of research. *Acad. Manag. Rev.* 25 217–226. 10.5465/amr.2000.2791611

[B137] Snyder C. R. Harris C. Anderson J. R. Holleran S. A. Irving L. M. Sigmon S. T. (1991). The will and the ways: development and validation of an individual-differences measure of hope. *J. Pers. Soc. Psychol.* 60 570–585. 10.1037/0022-3514.60.4.570 2037968

[B138] Soares A. S. Pais-Ribeiro J. L. Silva I. (2019). Developmental assets predictors of life satisfaction in adolescents. *Front. Psychol.* 10:236. 10.3389/fpsyg.2019.00236 30809171 PMC6379329

[B139] Stavros J. M. (1998). *Capacity Building: An Appreciative Approach.* Cleveland, OH: Case Western Reserve University.

[B140] Stavros J. M. (2020). Soar 2020 and beyond: strategy, systems innovation and stakeholder engagement. *AI Practitioner* 22 70–91. 10.12781/978-1-907549-43-4-11

[B141] Stavros J. M. Cole M. L. (2013). Soaring towards positive transformation and change. *ABAC ODI Vis. Act. Outcome* 1 10–34.

[B142] Stavros J. M. Hinrichs G. (2009). *Thinbook Of Soar: Creating Strengths-Based Strategy.* Bend, OR: Thin Book Publishers.

[B143] Stavros J. M. Saint D. (2010). “Soar: linking strategy and od to sustainable performance,” in *Practicing Organization Development: A Guide For Leading Change*, 3rd Edn, eds Rothwell W. Stavros J. Sullivan R. Sullivan A. (San Francisco, CA: Jossey-Bass), 377–394.

[B144] Stavros J. M. Torres C. (2018). *Conversations Worth Having: Using Appreciative Inquiry To Fuel Productive And Meaningful Engagement.* Oakland, CA: Berrett-Kohler Publishers, Inc.

[B145] Stavros J. M. Wooten L. (2012). “Positive strategy: creating and sustaining strengths-based strategy that soars and performs,” in *The Oxford Handbook Of Positive Organizational Scholarship*, eds Cameron K. S. Spreitzer G. M. (New York, NY: Oxford University Press), 824–842.

[B146] Stavros J. M. Cooperrider D. L. Kelley D. L. (2003). Strategic inquiry with appreciative intent: inspiration to SOAR! *AI Pract.* 5 10–17. 10.1108/14754390780000948

[B147] Steiner G. A. (1979). *Strategic Planning: What Every Manager Must Know.* New York, NY: Free Press.

[B148] Strange J. M. Mumford M. D. (2005). The origins of vision: effects of reflection, models, and analysis. *Leadersh. Q.* 16 121–148. 10.1016/j.leaqua.2004.07.006

[B149] Streiner D. L. (1994). Figuring out factors: the use and misuse of factor analysis. *Can. J. Psychiatry* 39 135–140. 10.1177/070674379403900303 8033017

[B150] Tedeschi R. G. Kilmer R. P. (2005). Assessing strengths, resilience, and growth to guide clinical interventions. *Prof. Psychol.* 36 230–237. 10.1037/0735-7028.36.3.230

[B151] Teece D. J. Pisano G. Shuen A. (1997). Dynamic capabilities and strategic management. *Strateg. Manag. J.* 18 509–533.

[B152] Thierry V. (1998). Results-oriented versus rules-oriented trade policies: a theoretical survey. *Eur. Econ. Rev.* 42 733–744. 10.1016/s0014-2921(97)00134-7

[B153] Tombaugh J. R. (2005). Positive leadership yields performance and profitability: effective organizations develop their strengths. *Dev. Learn. Org.* 19 15–17. 10.1108/14777280510590031

[B154] Tóth-Király I. Morin A. J. S. Bőthe B. Orosz G. Rigó A. (2018). Investigating the multidimensionality of need fulfillment: a bifactor exploratory structural equation modeling representation. *Struct. Equ. Modeling* 25 267–286. 10.1080/10705511.2017.1374867

[B155] Tóth-Király I. Morin A. J. S. Gillet N. Bőthe B. Nadon L. Rigó A. (2020). Refining the assessment of need supportive and need thwarting interpersonal behaviors using the bifactor exploratory structural equation modeling framework. *Curr. Psychol.* 1–15. 10.1007/s12144-020-00828-8

[B156] Trivette C. M. Dunst C. J. Deal A. G. Hamer A. W. Propst S. (1990). Assessing family strengths and family functioning style. *Topics Early Childhood Special Educ.* 10 16–35. 10.1177/027112149001000103

[B157] Van Zyl L. E. Ten Klooster P. M. (2022). Exploratory structural equation modeling: practical guidelines and tutorial with a convenient online tool for mplus. *Front. Psychiatry* 12:795672. 10.3389/fpsyt.2021.795672 35069293 PMC8779472

[B158] Vandenberg R. J. Lance C. E. (2000). A review and synthesis of the measurement invariance literature: suggestions, practices, and recommendations for organizational research. *Org. Res. Methods* 3 4–69. 10.1177/109442810031002

[B159] Verbeeten F. H. M. Speklé R. F. (2015). Management control, results-oriented culture and public sector performance: empirical evidence on new public management. *Org. Stud.* 36 953–978. 10.1177/0170840615580014

[B160] Verleysen B. Lambrechts F. Van Acker F. (2015). Building psychological capital with appreciative inquiry: investigating the mediating role of basic psychological need satisfaction. *J. Appl. Behav. Sci.* 51 10–35. 10.1177/0021886314540209

[B161] Wang J. Wang X. (2020). *Structural Equation Modelling: Applications Using Mplus*, 2nd Edn. Chichester: John Wiley & Sons Ltd.

[B162] Wang S. Chen C.-C. Dai C.-L. Richardson G. B. (2018). A call for, and beginner’s guide to, measurement invariance testing in evolutionary psychology. *Evol. Psychol. Sci.* 4 166–178. 10.1007/s40806-017-0125-5

[B163] Watkins M. W. (2018). Exploratory factor analysis: a guide to best practice. *J. Black Psychol.* 44 219–246. 10.1177/0095798418771807

[B164] Welch D. Grossaint K. Reid K. Walker C. (2014). Strengths-based leadership development: Insights from expert coaches. *Consult. Psychol. J.* 66 20–37. 10.1037/cpb0000002

[B165] Whitney D. D. Trosten-Bloom A. (2003). *The Power Of Appreciative Inquiry: A Practical Guide To Positive Change.* San Francisco, CA: Berrett-Koehler Publishers, Inc.

[B166] Wirtenberg J. Russell W. Lipsky D. (2017). *The Sustainable Enterprise Fieldbook: When It All Comes Together.* New York, NY: Routledge.

[B167] Wong C. S. Law K. S. (2002). The effects of leader and follower emotional intelligence on performance and attitude: an exploratory study. *Leadersh. Q.* 13 243–274. 10.1016/s1048-9843(02)00099-1

[B168] Wooten L. P. Crane P. (2004). Generating dynamic capabilities through a humanistic work ideology: the case of a certified-nurse midwife practice in a professional bureaucracy. *Am. Behav. Sci.* 47 848–866. 10.1177/0002764203260213

[B169] Zijlmans E. A. O. Tijmstra J. Van Der Ark L. A. Sijtsma K. (2019). Item-score reliability as a selection tool in test construction. *Front. Psychol.* 9:2298. 10.3389/fpsyg.2018.02298 30687144 PMC6336834

